# Apoptotic Cells Trigger Calcium Entry in Phagocytes by Inducing the Orai1-STIM1 Association

**DOI:** 10.3390/cells10102702

**Published:** 2021-10-09

**Authors:** Deokhwan Kim, Hyunji Moon, Hyeokjin Cho, Chanhyuk Min, Byeongjin Moon, Susumin Yang, Juyeon Lee, Sang-Ah Lee, Hyunjin Park, Dae-Hee Lee, Dongtak Jeong, Gwangrog Lee, Daeho Park

**Affiliations:** 1 School of Life Sciences, Gwangju Institute of Science and Technology, Gwangju 61005, Korea; po7322@gist.ac.kr (D.K.); hjmoon311@gm.gist.ac.kr (H.M.); jhj@gm.gist.ac.kr (H.C.); alscksgur@gist.ac.kr (C.M.); byeongjinmoon@gist.ac.kr (B.M.); susuminy@gist.ac.kr (S.Y.); iris260@gist.ac.kr (J.L.); sanga03@gist.ac.kr (S.-A.L.); malchic22@gist.ac.kr (H.P.); glee@gist.ac.kr (G.L.); 2 Center for Cell Mechanobiology, Gwangju Institute of Science and Technology, Gwangju 61005, Korea; 3 Department of Marine Food Science and Technology, Gangneung-Wonju National University, Gangneung 25456, Korea; neogene@gwnu.ac.kr; 4 Department of Molecular and Life Science, College of Science and Convergence Technology, Hanyang University ERICA Campus, Ansan 15588, Korea; cooljdt@hanyang.ac.kr

**Keywords:** efferocytosis, Orai1, STIM1, interaction, Mertk, SOCE, calcium flux

## Abstract

Swift and continuous phagocytosis of apoptotic cells can be achieved by modulation of calcium flux in phagocytes. However, the molecular mechanism by which apoptotic cells modulate calcium flux in phagocytes is incompletely understood. Here, using biophysical, biochemical, pharmaceutical, and genetic approaches, we show that apoptotic cells induced the Orai1-STIM1 interaction, leading to store-operated calcium entry (SOCE) in phagocytes through the Mertk-phospholipase C (PLC) γ1-inositol 1,4,5-triphosphate receptor (IP_3_R) axis. Apoptotic cells induced calcium release from the endoplasmic reticulum, which led to the Orai1-STIM1 association and, consequently, SOCE in phagocytes. This association was attenuated by masking phosphatidylserine. In addition, the depletion of Mertk, which indirectly senses phosphatidylserine on apoptotic cells, reduced the phosphorylation levels of PLCγ1 and IP_3_R, resulting in attenuation of the Orai1-STIM1 interaction and inefficient SOCE upon apoptotic cell stimulation. Taken together, our observations uncover the mechanism of how phagocytes engulfing apoptotic cells elevate the calcium level.

## 1. Introduction

Efferocytosis is a process that removes apoptotic cells generated throughout life in multicellular organisms and is essential for homeostasis and during development [1]. In this process, apoptotic cells are specifically removed by phagocytes through interactions between ligands on apoptotic cells and their receptors on phagocytes [2]. The best-known ligand on apoptotic cells is phosphatidylserine (PS), which is present on the inner leaflet of the plasma membrane in living cells and on the outer leaflet of the plasma membrane in apoptotic cells [3,4]. Phagocytes express two types of engulfment receptors that directly or indirectly bind to PS on apoptotic cells [5]. Tim-4, BAI1, and Stabilin-2, which are also called PS receptors, directly bind to PS on apoptotic cells [6,7,8], whereas TAM receptors and some integrins sense PS on apoptotic cells through the bridging molecules Gas6 and Mfge8, respectively [9].

The TAM receptors, Tyro3, Axl, and Mertk, comprise a unique family of receptor tyrosine kinases (RTKs). This family regulates various cellular processes, including cell proliferation, adhesion, migration, and efferocytosis [10]. The function of Mertk in efferocytosis in the retina and testis is well-established [11,12,13]. Mertk contains two Ig and two fibronectin type III domains located in its extracellular region and a kinase domain located in its cytosolic tail. The Ig domains of Mertk associate with Gas6, which binds to PS on apoptotic cells through its Gla domain, thereby promoting phagocytosis of apoptotic cells [14]. The kinase domain of Mertk is also important for efferocytosis because a Mertk mutant lacking this domain fails to promote engulfment of apoptotic cells [15]. In addition, apoptotic cell stimulation induces phosphorylation of Mertk and phospholipase C (PLC) γ2 and the association of these two proteins. These suggest that Mertk can transduce signals via its kinase domain and PLCγ2 during efferocytosis [16]. However, signal transduction downstream of Mertk during efferocytosis is incompletely understood.

Calcium is involved in a remarkably diverse array of cellular processes in which it functions as a second messenger during signal transduction. Due to its critical roles, the intracellular level of calcium is tightly regulated by various calcium channels and intracellular calcium stores, such as the endoplasmic reticulum (ER) and mitochondria [17,18]. One central mechanism regulating the intracellular calcium level is store-operated calcium entry (SOCE), which is mediated by Orai1, a calcium release-activated channel (CRAC), and STIM1, a calcium sensor in the ER. Depletion of calcium in the ER causes STIM1 to accumulate at ER-plasma membrane junctions, where it associates with and activates Orai1, which induces extracellular calcium entry though Orai1 [19,20]. Orai1 is typically activated by activation of G protein-coupled receptors or RTKs that activate PLC to cleave phosphatidylinositol 4,5-bisphosphate (PIP_2_) into inositol 1,4,5-triphosphate (IP_3_), which induces IP_3_ receptor (IP_3_R)-mediated calcium release from the ER [21]. Similar to other cellular processes, calcium is essential for efferocytosis, and its level is modulated for efficient efferocytosis. Thus, inhibition or deficiency of genes involved in calcium flux abrogates efferocytosis [22,23,24]. However, the molecular mechanism by which apoptotic cells modulate calcium flux in phagocytes remains elusive.

In this study, we found that apoptotic cell stimulation induced the Orai1-STIM1 association in phagocytes. This association was attenuated by masking PS on apoptotic cells, but not by blocking internalization or degradation of apoptotic cells. We further found that apoptotic cell stimulation augmented the phosphorylation of PLCγ1 and IP_3_R. However, this phosphorylation was weakened, and the Orai1-STIM1 association upon apoptotic cell stimulation was attenuated in *Mertk^−/−^* bone marrow-derived macrophages (BMDMs), leading to reduced calcium entry into phagocytes. Collectively, our observations suggest that apoptotic cells induce the Orai1-STIM1 association through the Mertk-PLCγ1-IP_3_R axis, triggering SOCE and elevation of the calcium level in phagocytes during efferocytosis.

## 2. Materials and Methods

### 2.1. Plasmids and Antibodies

All DNA constructs were generated by a PCR-based method and sequenced to confirm their fidelity. Orai1 and STIM1 were amplified from Orai1 (MMM1013-20276444), and STIM1 (MMM1013-202764946) cDNA purchased from Open Biosystems and introduced into pEBB vectors. For Orai1-CFP and STIM1-YFP vector construction, CFP and YFP were amplified from Raichu-Rac1 [25] and C-terminally introduced into pEBB-Orai1 and pEBB-STIM1, respectively. Anti-Flag (Sigma, F1804, St. Louis, MO, USA), anti-Orai1 (Santa Cruz, sc-68895, Dallas, TX, USA), anti-Orai1 (Abcam, ab111960, Cambridge, UK), anti-STIM1 (Abcam, ab108994), anti-IP_3_R (Cell Signaling, #8568, Boston, MA, USA), anti-phospho-IP_3_R (Cell Signaling, #3760S), anti-PLCγ1 (Cell Signaling, 2822S), anti-phospho-PLCγ1 (Cell Signaling, 2821S), anti-Mer (R&D systems, AF591, Minneapolis, MN, USA), and anti-β-Actin (Santa Cruz, sc-1616) were purchased.

### 2.2. Cell Culture and Transfection

LR73 cells were maintained in alpha-MEM containing 10% fetal bovine serum (FBS) and 1% penicillin-streptomycin-glutamine (PSQ). BMDMs were maintained in RPMI containing 10% L929-conditioned medium, 10% FBS, and 1% PSQ. NIH3T3 and RAW264.7 cells were maintained in DMEM containing 10% FBS and 1% PSQ. LR73 cells were transfected with plasmids using Lipofectamine 2000 (Invitrogen, Waltham, MA, USA) or Fugene HD (Promega, Madison, WI, USA), according to the manufacturer’s instructions.

### 2.3. Mice

*Mertk^−/−^* mice were purchased from Jackson laboratory. C57BL/6 were purchased from Taconic bioscience. Approximately 8~10-week-old mice were used for experiments regardless of sex. All experiments using mice were approved by the animal care and ethics committees of the Gwangju institute of science and technology (GIST) in accordance with the national institutes of health guide for the care and use of laboratory animals.

### 2.4. Binding and Internalization Assay

BMDMs plated on a cover glass in a 12-well non-culture plate were stained with 0.5 μM CellTracker (Thermo, C7025, Waltham, MA, USA) and then incubated with TAMRA-stained apoptotic thymocytes at 4 °C for 1 h in the presence or absence of calcium. After that, the cells were washed with PBS and fixed with 4% PFA. To measure the effect of extracellular calcium on internalization of apoptotic cells, CellTracker-stained BMDMs were incubated with pHrodo-labeled apoptotic thymocytes at 4 °C for 1 h. After that, unbound apoptotic cells were removed by PBS washing, and the BMDMs were further incubated at 37 °C for 30 min in the presence or absence of Ca^2+^. Images were acquired using Axio Imager D2 (Zeiss, Jena, Germany).

### 2.5. Measurement of Intracellular Calcium

LR73, NIH3T3, RAW264.7, and BMDMs were stained with 1 μM Fluo4-AM (Thermo, F14201, green) in staining buffer (10 mM HEPES, 150 mM NaCl, 5 mM KCl, 0.1% glucose, 1% FBS, 2.5 mM probenecid, 1 mM CaCl_2_, 1 mM MgCl_2_, and pH 7.4) at 37 °C for 30 min, pretreated with 10~200 μM 2-APB (Tocris Bioscience, 1224, Minneapolis, MN, USA), 20 μM nifedipine (Sigma, N7634), 50 μM SKF-96365 (Sigma, S7809), or 100 μM YM58483 (Sigma, Y4895) and incubated with TAMRA-stained apoptotic thymocytes in the presence of the indicated concentrations of the inhibitors for 10 min. Then, the cells were analyzed by flow cytometry (BD FACS Canto II).

### 2.6. Engulfment Assay

Engulfment assay was performed as previously described [26]. LR73 cells or BMDMs derived from the indicated mice were incubated with TAMRA-stained apoptotic thymocytes for 2 h or 30 min, respectively. Then, the cells were extensively washed with PBS to remove unbound apoptotic cells, trypsinized, suspended, and analyzed by flow cytometry (BD FACS Canto II). TAMRA-positive phagocytes were considered phagocytes engulfing apoptotic cells. To evaluate the effects of inhibitors on efferocytosis, BMDMs were pre-incubated with 10 mM EGTA, 10~200 μM 2-APB (Tocris Bioscience, 1224), 20 μM Nifedipine (Sigma, N7634), 100 μM YM58483 (Sigma, Y4895), 1 μM Cytochalasin D (Sigma, C8273), 50 μM SKF-96365 (Sigma, S7809) or 1 μM Bafilomycin A (Abcam, ab120497) for 30 min (BMDMs) or 2 h (LR73 cells), and then the engulfment assay was performed.

### 2.7. Immunoblotting and Immunoprecipitation

LR73 cells transfected with the indicated plasmids or BMDMs were incubated with apoptotic cells for 10 min and then lysed using lysis buffer (50 mM Tris (pH 7.6), 150 mM NaCl, 10 mM NaPP, 10 mM NaF, 1 mM Na_3_VO_4_, 1% Triton X-100, 10 μg/mL pepstatin, 10 μg/mL leupeptin, 10 μg/mL AEBSF, and 10 μg/mL aprotinin). Proteins in the lysates were separated by SDS-PAGE, transferred onto a nitrocellulose membrane, and detected by appropriate antibodies. For immunoprecipitation, the lysates of cells were incubated with anti-FLAG conjugated-agarose beads (Sigma, A2220), glutathione-sepharose 4B beads (GE healthcare, 17-0756-01, Boston, MA, USA), or protein A/G agarose beads (Santa Cruz, sc-2003) with appropriate antibodies for 2 h. Proteins bound to the beads were detected by immunoblotting. ImageJ was used to quantify band intensities.

### 2.8. FRET Analysis for Orai1-STIM1 Association

LR73 cells plated on confocal dishes were transfected with Orai1-CFP and STIM1-YFP. One day after transfection, the cells were stimulated with TAMRA-stained apoptotic cells for the indicated duration, and then the cells expressing both Orai1-CFP and STIM1-YFP were observed. FRET images were obtained every 10 sec with the ratio imaging method equipped with Olympus FV1c000 SPD (Olympus, Tokyo, Japan). For FRET ratio calculation, CFP and YFP images were subtracted with respective background intensity calculated by the mean intensity value of pixels inside the background mask. The FRET ratio was calculated as the ratio between processed YFP and CFP images. The FRET ratio was presented as a heatmap by mapping the FRET ratio to color and total intensity to opacity. All processing was performed, using custom code of MATLAB R2019b and image processing toolbox. To measure FRET using a microplate reader (FlexStation 3, Molecular devices, San Jose, CA, USA), LR73 cells plated in a 96-well culture plate were transfected with Orai1-CFP and STIM1-YFP. One day after transfection, the cells were stimulated with apoptotic or live thymocytes. The FRET values were measured every 10 s by the microplate reader.

### 2.9. SOCE

BMDMs derived from *WT* or *Mertk^−/−^* were plated in a 96-well culture plate and stained with Fluo4-AM (Thermo, F14201, green) in staining buffer (10 mM HEPES, 150 mM NaCl, 5 mM KCl, 0.1% glucose, 1% FBS, 2.5 mM probenecid, 1 mM CaCl_2_, 1 mM MgCl_2_, and pH 7.4). The phagocytes were treated with 0.1 µM thapsigargin (Sigma, T9033) for the indicated duration. After that, apoptotic cells or live cells in RPMI containing 1.0 mM calcium were added to the phagocytes at the indicated time. Note that 0.1 µM thapsigargin and 1.0 mM calcium were used. The intensity of fluorescence from the cells was measured using a microplate reader (FlexStation 3, Molecular Devices). The peak of SOCE is the maximal fluorescence of Fluo4. Slope is (Fmax − F360s)/(ta − t360s), where ta is the time value at which the fluorescence of Fluo4 is maximal.

### 2.10. Statistical Analysis

Data are shown as the mean ± standard error of mean (SEM). All experiments were independently performed at least 3 times. Statistical significance of difference was analyzed by unpaired Student’s two-tailed *t* test, one-way ANOVA, or two-way ANOVA, using GraphPad Prism 7. A *p* value of <0.05 was considered to be statistically significant.

## 3. Results

### 3.1. Extracellular Calcium Is Required for Internalization of Apoptotic Cells

**Figure 1:**
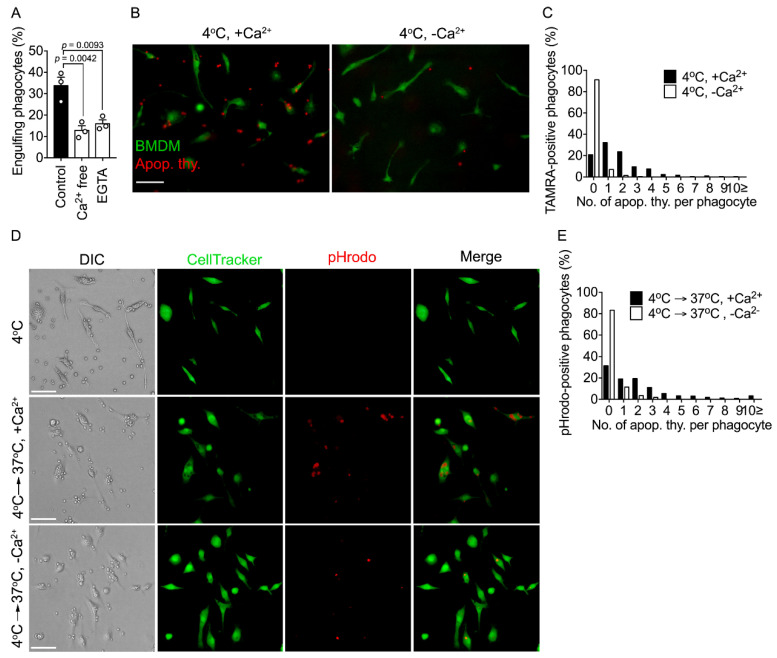
Extracellular calcium is necessary for internalization of apoptotic cells. (**A**) BMDMs treated with EGTA (10 mM) or cultured in calcium-free DMEM were incubated with TAMRA-stained apoptotic thymocytes for 30 min and analyzed by flow cytometry. TAMRA-positive BMDMs were considered to be phagocytes engulfing apoptotic cells. Control BMDMs incubated with apoptotic cells in DMEM containing calcium. n = 3 experiments, mean ± SEM (one-way ANOVA). (**B**,**C**) CellTracker-stained BMDMs were incubated with TAMRA-labeled apoptotic thymocytes at 4 °C for 1 h in the presence or absence of calcium and observed by microscopy (**B**). The number of apoptotic cells bound to phagocytes was quantified (**C**). Scale bar, 50 µm. n = 292 (+Ca^2+^), 283 (−Ca^2+^) cells. (**D**,**E**) CellTracker-stained BMDMs were incubated with pHrodo-labeled apoptotic thymocytes at 4 °C for 1 h, washed with PBS to remove unbound apoptotic thymocytes, and further incubated at 37 °C for 30 min in the presence or absence of calcium (**D**). The number of pHrodo-positive BMDMs was quantified (**E**). Scale bar, 100 µm. n = 400 cells.

Both extra- and intracellular calcium are essential for efferocytosis; however, the reasons why are incompletely understood. Calcium is necessary for binding of PS to its receptors [27,28,29]; therefore, it is possible that extracellular calcium is crucial for recognition and engulfment of apoptotic cells by phagocytes. We confirmed this hypothesis. Phagocytosis of apoptotic cells by BMDMs treated with EGTA or incubated in calcium-free medium was drastically diminished (Figure 1A), which was likely because apoptotic cells did not bind to them well (Figure 1B,C). However, it is uncertain whether extracellular calcium is solely required for recognition of apoptotic cells by phagocytes. To investigate this, BMDMs were allowed to bind to apoptotic cells without internalization by incubation at 4 °C and then incubated at 37 °C in the presence or absence of calcium. Phagocytes incubated in the presence of calcium engulfed apoptotic cells, whereas phagocytes incubated in the absence of calcium bound to, but engulfed few, apoptotic cells (Figure 1D,E). These data suggest that extracellular calcium is required for other stages of efferocytosis following binding of apoptotic cells to phagocytes, implying that it enters phagocytes.

### 3.2. Elevation of the Calcium Level in Phagocytes Is Due to Extracellular Calcium Entry during Efferocytosis

**Figure 2:**
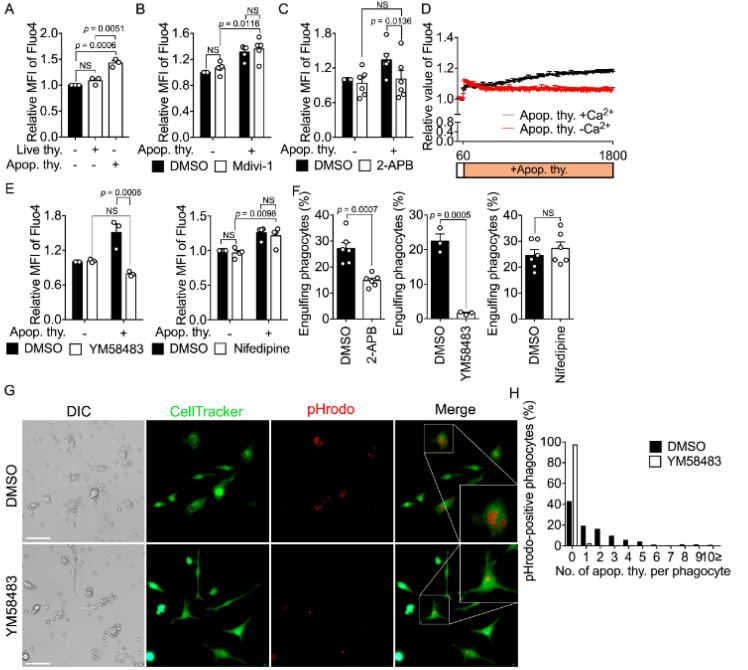
Elevation of the calcium level in phagocytes is due to extracellular calcium entry during efferocytosis (**A**) Fluo4-stained BMDMs were incubated with apoptotic thymocytes or live thymocytes and analyzed by flow cytometry. The MFIs of Fluo4 in BMDMs were compared. n = 3 experiments, mean ± SEM (one-way ANOVA). (**B**,**C**) Fluo4-stained BMDMs were incubated with apoptotic cells in the presence or absence of Mdivi-1 (10 µM) (**B**, n = 5 experiments) or 2-APB (200 µM) (**C**, n = 6 experiments). The MFIs of Fluo4 in BMDMs were measured by flow cytometry. Mean ± SEM. NS, not significant (two-way ANOVA). (**D**) Fluo4-stained BMDMs were incubated with apoptotic cells in DMEM or calcium-free DMEM. The fluorescence intensity of Fluo4 in BMDMs was measured using FlexStation. n = 4 experiments, mean ± SEM. (**E**) Fluo-4-stained BMDMs were incubated with apoptotic cells in the presence or absence of YM58483 (100 µM, n = 3 experiments) or Nifedipine (20 µM, n = 4 experiments) and analyzed by flow cytometry. Mean ± SEM (two-way ANOVA). (**F**) BMDMs were incubated with TAMRA-stained apoptotic cells for 30 min in the presence or absence of 2-APB (200 µM), YM58483 (100 µM), or Nifedipine (20 µM) and analyzed by flow cytometry. n = 6 (left), n = 3 (middle), and n = 6 (right) experiments, mean ± SEM (two-tailed unpaired Student’s *t* test). (**G**,**H**) CellTracker-stained BMDMs were incubated with pHrodo-stained apoptotic thymocytes for 30 min in the presence or absence of YM58483 (100 µM) and observed by microscopy (**G**) and the number of pHrodo-positive apoptotic cells per phagocyte was quantified (**H**). Insets are enlargements of phagocytes engulfing apoptotic cells. Scale bar, 100 μM.

The calcium level in phagocytes increases during efferocytosis. This is consistent with our extended observations, using various types of phagocytes, including professional and non-professional phagocytes and using Fura-2, a ratiometric dye (Figure 2A and Figure S1A–D). Based on the finding that extracellular calcium is necessary for later stages of efferocytosis following the binding of apoptotic cells, elevation of the intracellular calcium level during efferocytosis may be due to extracellular calcium entry. However, other mechanisms, such as calcium release from intracellular stores and/or decreased calcium uptake by mitochondria, may underlie elevation of the intracellular calcium level. We first investigated whether decreased mitochondrial calcium uptake underlies elevation of the intracellular calcium level during efferocytosis, using Mdivi-1, which blocks mitochondrial fission through Drp-1 and thus promotes mitochondrial calcium uptake through the mitochondrial calcium uniporter (MCU) [30]. Mdivi-1 did not significantly alter the calcium level in BMDMs incubated without or with apoptotic cells (Figure 2B), suggesting that mitochondrial calcium flux is not a major contributor to elevation of the intracellular calcium level during efferocytosis. We next tested whether calcium release from the ER underlies elevation of the intracellular calcium level during efferocytosis, using 2-APB. It blocks IP_3_R-mediated calcium release from the ER with an additional inhibitory effect on SOCE [31,32]. 2-APB abolished the increase in the calcium level in BMDMs incubated with apoptotic cells (Figure 2C and Figure S2A), implying that calcium release from the ER likely is involved in elevation of the intracellular calcium level during efferocytosis. However, there is a possibility that the effect of 2-APB on the intracellular calcium level may be still caused by inhibiting SOCE in this experiment. Inhibition of IP_3_R can also block calcium entry into cells because calcium release from the ER activates CRACs and thus induces calcium entry through these channels. In addition, calcium may enter phagocytes through other channels, such as voltage-gated calcium channels during efferocytosis. To investigate this, we incubated phagocytes with apoptotic cells in calcium-free medium and measured the intracellular calcium level. The calcium level in BMDM incubated with apoptotic cells in the presence of extracellular calcium continuously increased, whereas this phenomenon was not observed in the absence of extracellular calcium (Figure 2D), suggesting that elevation of the intracellular calcium level during efferocytosis is due to extracellular calcium entry. We next tested which type of calcium channels is involved in the elevation of the intracellular calcium level using calcium channel blockers. YM58483 and SKF-96365, SOCE and CRAC inhibitors, nullified the effect of apoptotic cells on the calcium level in phagocytes, but nifedipine, a voltage-dependent calcium channel inhibitor, did not (Figure 2E and Figure S2B). Noticeably, the effects of the inhibitors on elevation of the intracellular calcium level during efferocytosis corresponded to their effects on efferocytosis. 2-APB, YM58483, and SKF-96365 impeded efferocytosis, but nifedipine did not (Figure 2F and Figure S2C,D). Interestingly, YM58483 abrogated efferocytosis but did not affect the binding of apoptotic cells to phagocytes (Figure 2G,H), supporting the idea that extracellular calcium entry is necessary for later stages of efferocytosis following the binding of apoptotic cells (Figure 1D,E). In summary, these data suggest that calcium release from the ER and extracellular calcium entry caused by this contribute to elevation of the intracellular calcium level and are necessary for efferocytosis.

### 3.3. Apoptotic Cell Stimulation Induces the Orai1-STIM1 Interaction in Phagocytes

**Figure 3:**
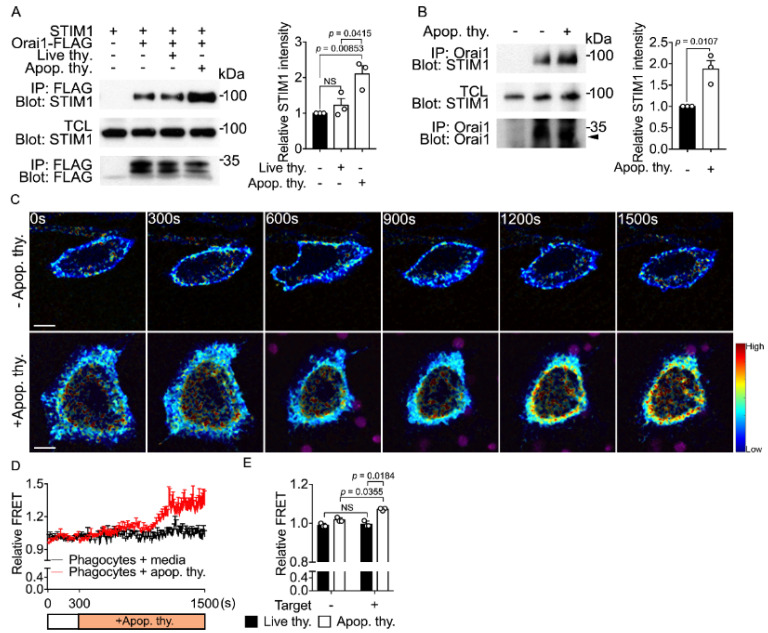
Apoptotic cell stimulation induces the Orai1-STIM1 interaction in phagocytes (**A**) LR73 cells were transfected with the indicated plasmids. After 1 day, cells were incubated with apoptotic thymocytes or live thymocytes for 10 min and lysed. FLAG-tagged Orai1 was immunoprecipitated with anti-FLAG antibody-conjugated agarose beads. Bound proteins were detected with the indicated antibodies (left) and co-immunoprecipitated STIM1 with Orai1 was quantified (right). The images are representative of three independent experiments. Mean ± SEM (two-tailed unpaired Student’s *t* test). (**B**) Lysates of BMDMs stimulated with apoptotic cells for 10 min were incubated with an anti-Orai1 antibody and protein A/G agarose beads. Bound proteins were detected by immunoblotting (left) and co-immunoprecipitated STIM1 with Orai1 was quantified (right). The arrow head indicates Orai1. The images are representative of three independent experiments. Mean ± SEM (two-tailed unpaired Student’s *t* test). (**C**,**D**) LR73 cells transfected with Orai1-CFP and STIM1-YFP were incubated with TAMRA-stained apoptotic cells for the indicated duration. FRET signals were observed by time-lapse confocal microscopy (**C**) and quantified (**D**). Images were obtained every 10 sec for 30 min. Scale bar, 10 µm. n = 12 (−Apop. thy) and n = 13 (+Apop. thy.) cells, mean ± SEM. (**E**) LR73 cells transfected with Orai1-CFP and STIM1-YFP were stimulated with apoptotic or living thymocytes. FRET signals were detected using a microplate reader. n = 3 experiments, mean ± SEM (two-way ANOVA).

Depletion of calcium from the ER is sensed by STIM1, which associates with and thereby activates Orai1, resulting in extracellular calcium entry through Orai1, a process called SOCE [19]. In addition, Orai1 and STIM1 are crucial for efferocytosis [22,23,24]. Thus, the association of Orai1 and STIM1 is likely modulated during efferocytosis. To investigate this, LR73 cells overexpressing STIM1 and Orai1 were stimulated with apoptotic cells or live cells and the Orai1-STIM1 association was monitored by an immunoprecipitation assay. Apoptotic cells increased Orai1-STIM1 association but not live cells (Figure 3A). This phenomenon was also observed at physiological protein levels. Orai1 and STIM1 associated more in BMDMs stimulated with apoptotic cells than in control BMDMs (Figure 3B). This increased association may be due to contamination of apoptotic cells. To investigate this and to observe the Orai1-STIM1 association in real time, we performed fluorescence resonance energy transfer (FRET) analysis. Orai1 and STIM1 C-terminally tagged with CFP and YFP, respectively, were introduced into LR73 cells and energy transfer was measured. FRET was ~35% higher in phagocytes stimulated with apoptotic cells than in control phagocytes (Figure 3C,D, and Video S1). Noticeably, FRET measured using a microplate reader was increased in phagocytes incubated with apoptotic cells, but not in those incubated with living cells (Figure 3E). These data suggest that apoptotic cells induce the Orai1-STIM1 association in phagocytes during efferocytosis.

### 3.4. Recognition of PS Is Necessary for Induction of the Orai1-STIM1 Association by Apoptotic Cells

**Figure 4:**
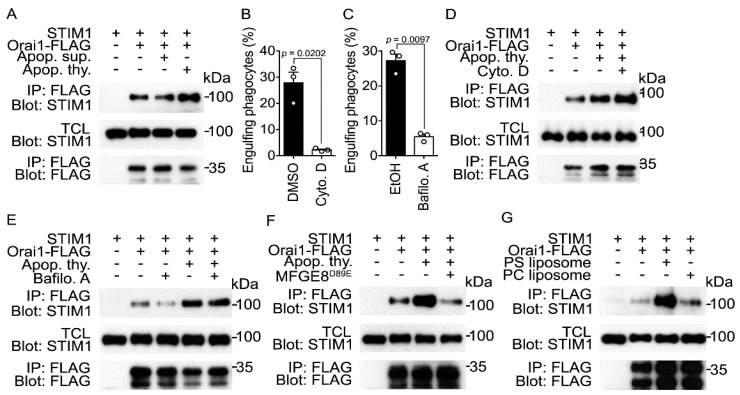
Apoptotic cell stimulation induces the Orai1-STIM1 association in a PS-dependent manner (**A**) LR73 cells transfected with the indicated plasmids were incubated with apoptotic cells or apoptotic cell supernatants for 10 min. Orai1-FLAG in cell lysates was precipitated with anti-FLAG antibody-conjugated agarose beads. Bound proteins were detected by immunoblotting. (**B**,**C**) LR73 cells were incubated with TAMRA-stained apoptotic cells for 2 h in the presence of the indicated concentration of cytochalasin D (1 µM) (**B**) or bafilomycin A (1 µM) (**C**), and engulfing phagocytes were analyzed by flow cytometry. n = 3 experiments, mean ± SEM (two-tailed unpaired Student’s *t* test). (**D**–**F**) LR73 cells transfected with the indicated plasmids were stimulated with apoptotic cells for 10 min in the presence or absence of cytochalasin D (1 µM) (**D**), bafilomycin A (1 µM) (**E**), or Mfge8^D89E^ (**F**). The Orai1-STIM1 association was detected as in (**A**). (**G**) LR73 cells transfected with Orai1 and STIM1 were stimulated with PC or PS liposomes for 10 min. Cell lysates were incubated with anti-FLAG antibody-conjugated agarose beads. Bound proteins were detected with the indicated antibodies. The images are representative of at least three independent experiments (**A**,**D**–**G**).

Next, we dissected the molecular mechanism by which apoptotic cells induce the Orai1-STIM1 association. First, we investigated whether apoptotic cells themselves or factors secreted by them induce the Orai1-STIM1 association. The Orai1-STIM1 association was not induced by apoptotic cell supernatants but was robustly induced by apoptotic cells (Figure 4A and Figure S3A). This indicates that apoptotic cells themselves, but not molecules secreted by them, are responsible for induction of the Orai1-STIM1 association and that binding of apoptotic cells to phagocytes and/or subsequent stages of efferocytosis, such as internalization, and degradation of apoptotic cells are necessary for induction of this association. Next, to address which phase of efferocytosis is important for induction of the Orai1-STIM1 association, the internalization and degradation of apoptotic cells were blocked by cytochalasin D, which inhibits actin polymerization, and bafilomycin A, which impedes lysosomal acidification and thus degradation of cargos, respectively. Cytochalasin D and bafilomycin A appreciably impeded efferocytosis (Figure 4B,C). However, the Orai1-STIM1 association in phagocytes incubated with apoptotic cells in the presence of these inhibitors was comparable with that in control phagocytes (Figure 4D,E and Figure S3B,C). This indicates that internalization and degradation of apoptotic cells are dispensable and binding of apoptotic cells to phagocytes is sufficient for induction of the Orai1-STIM1 association.

PS exposed on apoptotic cells is the best-known ligand to be directly or indirectly recognized by engulfment receptors on phagocytes. We thus tested whether PS is crucial for induction of the Orai1-STIM1 association during efferocytosis. To this end, PS on apoptotic cells was masked, using a Mfge8 mutant called Mfge8^D89E^, which binds to PS on apoptotic cells but not to integrins on phagocytes [33], and the Orai1-STIM1 association was measured upon addition of PS-masked apoptotic cells. Apoptotic cells pretreated with purified Mfge8^D89E^ failed to increase the Orai1-STIM1 association in phagocytes (Figure 4F and Figure S3D). This finding was replicated when PS on apoptotic cells was masked by Anxa5, a PS-binding protein (Figure S4), suggesting that PS on apoptotic cells is necessary for induction of the Orai1-STIM1 association during efferocytosis. To further investigate whether PS is sufficient to induce the Orai1-STIM1 association, phagocytes were incubated with PS liposomes, a simplified mimic of apoptotic cells. The Orai1-STIM1 association was augmented in phagocytes incubated with PS liposomes but was unaltered in phagocytes incubated with phosphatidylcholine (PC) liposomes (Figure 4G and Figure S3E). These data indicate that PS exposed on apoptotic cells is necessary and sufficient for induction of the Orai1-STIM1 association during efferocytosis. They further suggest that engulfment receptors that directly or indirectly recognize PS are upstream of signaling that induces the Orai1-STIM1 association.

### 3.5. Mertk Is an Upstream Receptor of the PLCγ1-IP_3_R Axis Activated by Apoptotic Cells

**Figure 5:**
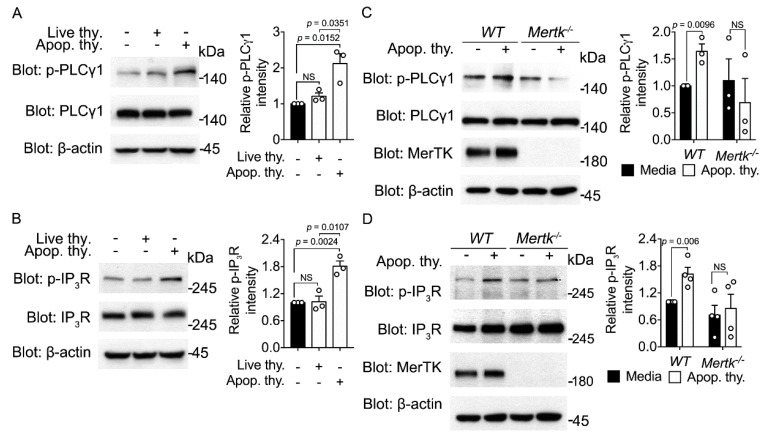
Apoptotic cell stimulation activates the PLCγ1-IP_3_R axis (**A**,**B**) BMDMs were incubated with apoptotic thymocytes or live thymocytes for 10 min and lysed. Phosphor-PLCγ1 (**A**) and phosphor-IP_3_R (**B**) in the lysates were detected by immunoblotting and quantified. β-actin was used as a loading control. The images are representative of three independent experiments. Mean ± SEM (two-tailed unpaired Student’s *t* test). (**C**,**D**) BMDMs derived from *Mertk^−/−^* and *WT* mice were incubated with apoptotic cells for 10 min and lysed. Phospho-PLCγ1 (**C**) and phosphor-IP_3_R (**D**) in the lysates were detected by immunoblotting and quantified. The images are representative of three (**C**) or four (**D**) independent experiments. Mean ± SEM (two-tailed unpaired Student’s *t* test).

A key signaling pathway for activation of Orai1 and induction of the Orai1-STIM1 association resulting in SOCE involves activation of PLC to cleave PIP_2_ into IP_3_ via G proteins or RTK cascades. IP_3_ then induces IP_3_R-mediated calcium release from the ER, which triggers the Orai1-STIM1 association and calcium entry through Orai1 [34]. Thus, we tested whether the PLCγ-IP_3_R axis is activated during efferocytosis by measuring the phosphorylation levels of PLCγ1 and IP_3_R. The levels of phosphorylated PLCγ1 and IP_3_R were higher in BMDMs incubated with apoptotic cells than in BMDMs incubated with live cells (Figure 5A,B), suggesting that the PLCγ1-IP_3_R axis is activated during efferocytosis and that an engulfment receptor is upstream of this axis.

Mertk is a member of the TAM receptor kinase family and also functions as an engulfment receptor that indirectly senses PS exposed on apoptotic cells through Gas6 and Pros [35]. In addition, PLCγ2 is recruited to Mertk upon apoptotic cell stimulation [16]. Thus, Mertk may be an engulfment receptor upstream of the PLCγ-IP_3_R axis that induces the Orai1-STIM1 association during efferocytosis. To investigate this, the phosphorylation levels of PLCγ1 and IP_3_R in BMDMs derived from *Mertk^−/−^* and wild-type (*WT*) mice were compared upon apoptotic cell stimulation. In the basal state, the total and phosphorylation levels of PLCγ1 and IP_3_R were comparable in *Mertk^−/−^* and *WT* BMDMs. However, the phosphorylation levels of PLCγ1 and IP_3_R were substantially lower in *Mertk^−/−^* BMDMs than in *WT* BMDMs upon incubation with apoptotic cells (Figure 5C,D), suggesting that Mertk is an upstream receptor of the PLCγ1-IP_3_R axis that induces the Orai1-STIM1 association.

### 3.6. Mertk Depletion Attenuates the Orai1-STIM1 Association and Calcium Entry during Efferocytosis

**Figure 6:**
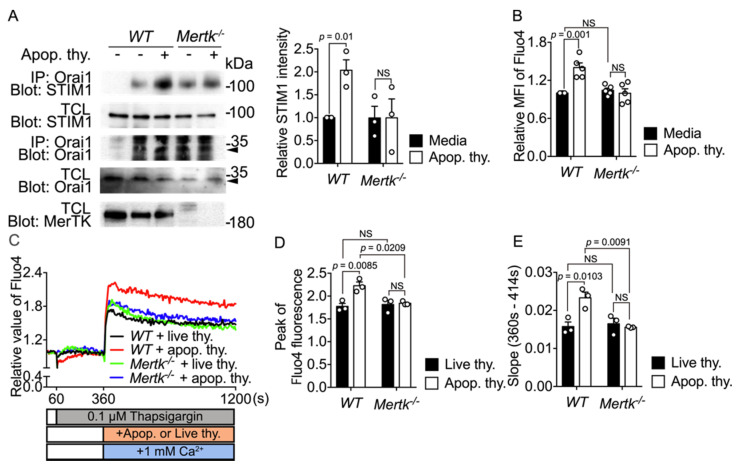
Mertk depletion attenuates the Orai1-STIM1 association and calcium entry (**A**) BMDMs derived from *Mertk^−/−^* and *WT* mice were incubated with apoptotic cells for 10 min. Cell lysates were incubated with an anti-Orai1 antibody and protein A/G-conjugated agarose beads. Bound proteins were detected with the indicated antibodies (left) and co-immunoprecipitated STIM1 with Orai1 was quantified (right). The arrow heads indicate Orai1. The images are representative of three independent experiments. Mean ± SEM (two-tailed unpaired Student’s *t* test). (**B**) BMDMs derived from *Mertk^−/−^* and *WT* mice were stained with Fluo4 and incubated with apoptotic cells. The MFIs of Fluo4 in the cells were analyzed by flow cytometry. n = 5 experiments, mean ± SEM (two-way ANOVA). (**C**–**E**) BMDMs from the indicated mice were stained with Fluo4 and then treated with 0.1 µM thapsigargin for the indicated duration. Thereafter, apoptotic or live thymocytes in medium containing 1.0 mM calcium were added to the cells at the indicated time. Fluorescence of the cells was measured with a microplate reader. Data are representative of 4 independent experiments (**C**), and the peak and slope of SOCE were calculated (**D**,**E**). n = 3 experiments, mean ± SEM (two-way ANOVA).

Next, to test whether Mertk functions as an upstream engulfment receptor that mediates the Orai1-STIM1 association during efferocytosis, this association was compared between *Mertk^−/−^* and *WT* BMDMs upon apoptotic cell stimulation. Orai1 and STIM1 associated substantially less in *Mertk^−/−^* BMDMs than in *WT* BMDMs following apoptotic cell stimulation (Figure 6A). Next, we tested whether attenuation of the Orai1-STIM1 association in *Mertk^−/−^* BMDMs upon apoptotic cell stimulation was coupled with the intracellular calcium level. The basal calcium level was comparable in *Mertk^−/−^* and *WT* BMDMs. However, apoptotic cell stimulation failed to increase the calcium level in *Mertk^−/−^* BMDMs (Figure 6B), suggesting that Mertk is an upstream receptor that elevates the intracellular calcium level during efferocytosis. We then tested whether the inability of apoptotic cell stimulation to increase the calcium level in *Mertk^−/−^* BMDMs is due to alteration of SOCE. To this end, calcium in the ER was depleted by thapsigargin and calcium entry was monitored upon adding apoptotic cells. Intrinsic SOCE was indistinguishable between *Mertk^−/−^* and *WT* BMDMs. However, *Mertk^−/−^* BMDMs were unable to further increase SOCE upon apoptotic cell stimulation but *WT* BMDMs did (Figure 6C). SOCE, represented by the peak of Fluo4 fluorescence, was increased by 19%, and the rate of calcium influx, as indicated by the slope (360–414 s), was also significantly increased in *WT* BMDMs. However, these phenomena were not observed in *Mertk^−/−^* BMDMs upon apoptotic cell stimulation (Figure 6D,E), suggesting that Mertk is necessary for calcium entry during efferocytosis. Taken together, these results show that the Orai1-STIM1 association is induced through the PLCγ1-IP_3_R axis downstream of Mertk, resulting in calcium entry and eventually elevation of the calcium level in phagocytes during efferocytosis.

## 4. Discussion

Efferocytosis is a sophisticatedly regulated process that specifically removes dying cells. Both intra- and extracellular calcium are indispensable for this essential process. In this study, we found that extracellular calcium enters phagocytes to increase the intracellular calcium level during efferocytosis, and this is mediated by the Orai1-STIM1 association induced by the Mertk-PLCγ1-IP_3_R axis. A variety of engulfment receptors, including PS receptors, require calcium for binding to PS on apoptotic cells, and this may be one reason why extracellular calcium is necessary for efferocytosis. Our observations suggest that extracellular calcium also supplements intracellular calcium during efferocytosis. However, the roles of calcium in efferocytosis after it enters phagocytes remain unclear. Indeed, few studies have shown how calcium functions in efferocytosis. Recently, our group reported that Crbn, which is a component of the CRL4^CRBN^ E3 ligase and regulates the level of Orai1, alters the amount of time required to internalize apoptotic cells [22]. Thus, one plausible role of calcium during efferocytosis is appropriate closure of the phagocytic cup to internalize apoptotic cells. We are currently studying the phases of efferocytosis in which calcium is involved.

The ER and mitochondria are intracellular organelles that store calcium [36]. A previous study reported that the upregulation of Drp-1 during efferocytosis increases mitochondrial fission, which blocks MCU-mediated mitochondrial calcium uptake and thus augments the intracellular calcium level [30]. Initially, we thought that this mechanism also augments the intracellular calcium level during efferocytosis. However, Mdivi-1 did not inhibit elevation of the intracellular calcium level, although it somewhat impeded efferocytosis. This discrepancy might be due to differences in the experimental conditions, such as the cell type used and the methods employed to measure the intracellular calcium level. Indeed, GCaMP6f, which was used as a calcium indicator in the previous study, may detect calcium more sensitively than Fluo4, which was used in this study. Thus, an inhibitory effect of Mdivi-1 on elevation of the intracellular calcium level might not have been detected. Nevertheless, we clearly showed that elevation of the intracellular calcium level upon apoptotic cell stimulation was abrogated in the presence of an CRAC inhibitor and in a calcium-free medium. This suggests that elevation of the intracellular calcium level during efferocytosis is mainly due to extracellular calcium entry and that diminished mitochondrial calcium uptake seems to only contribute marginally, if at all.

Apoptotic cell stimulation induces the Orai1-STIM1 association in a PS-dependent manner. The roles of Tim-4, a PS receptor, in efferocytosis are well-characterized [7,37]. Tim-4 cooperates with Mertk and integrins to phagocytose apoptotic cells [38,39,40]. Therefore, we analyzed the effect of Tim-4 depletion on elevation of the intracellular calcium level upon apoptotic cell stimulation. In contrast with *Mertk^−/−^* phagocytes, the intracellular calcium level was comparable in *Tim-4^−/−^* and *WT* peritoneal macrophages upon apoptotic cell stimulation (data not shown). This may be because Tim-4 does not mediate direct signaling [41,42] and the experiments were performed in the presence of serum, which enabled Mertk to sufficiently recognize apoptotic cells itself. In addition, we showed that intrinsic SOCE in *Mertk^−/−^* and *WT* BMDMs is comparable. However, when SOCE was induced by 1 µM thapsigargin and 2 mM calcium, a typical condition to measure intrinsic SOCE, SOCE in *Mertk^−/−^* BMDMs was lower than in *WT* BMDMs. Apoptotic cell stimulation failed to increase SOCE in both *WT* and *Mertk^−/−^* BMDMs at this condition (data not shown). It may be because the thapsigargin concentration produces a stimulus inducing maximal SOCE. Nevertheless, the reason why intrinsic SOCE is lower in *Mertk^−/−^* phagocytes than in *WT* phagocytes remains unclear at this condition and it would be interesting to investigate this in the future.

Collectively, the data presented in this study suggest that induction of the Orai1-STIM1 association during efferocytosis increases the calcium level in phagocytes through SOCE and that Mertk is upstream of the PLCγ1-IP_3_R axis responsible for induction of this association. Thus, our findings may help to comprehensively understand calcium flux in efferocytosis and to develop therapeutics for diseases associated with efferocytosis.

## Data Availability

Data supporting the findings of the study are available within the article and Supplementary Materials or from the corresponding author upon reasonable request.

## Supplementary Materials

The following are available online at https://www.mdpi.com/article/10.3390/cells10102702/s1, Figure S1: Elevation of the calcium level in phagocytes during efferocytosis, Figure S2: The effects of 2-APB and SKF-96365 on efferocytosis and the calcium elevation in phagocytes, Figure S3: Quantification of immunoblots in Figure 4, Figure S4: Blocking PS on apoptotic cells attenuates Orai1-STIM1 association, Video S1: apoptotic cell stimulation induces Orai1-STIM1 interaction.
