# Supplementary material for: Apoptotic Cells Trigger Calcium Entry in Phagocytes by Inducing the Orai1-STIM1 Association
Source: Cells. 2021 Oct 9;10(10):2702. doi: 10.3390/cells10102702 (PMC8534458; doi:10.3390/cells10102702)
Supplement: Supplementary file 1 [file cells-10-02702-s001.zip › Supplementary Figures_Cells_revised.pdf]

Supplementary Figures

Apoptotic cells trigger calcium entry in phagocytes during efferocytosis by inducing the Orai1-STIM1 association

Deokhwan Kim[1,2], Hyunji Moon[1,2], Hyeokjin Cho[1,2], Chanhyuk Min[1,2], Byeongjin Moon[1,2], Susumin Yang[1,2], Juyeon Lee[1, 2], Sang-Ah Lee[1,2], Hyeonjin Park[1,2], Dae-Hee Lee[3], Dongtak Jeong[4], Gwangrog Lee[1, 2], Daeho Park[1, 2, *]

1. School of Life Sciences, Gwangju Institute of Science and Technology, Gwangju 61005, Korea

2. Center for Cell Mechanobiology, Gwangju Institute of Science and Technology, Gwangju 61005, Korea

3. Department of Marine Food Science and Technology, Gangneung-Wonju National University, Gangneung 25456, Korea

4. Department of Molecular and Life Science, College of Science and Convergence Technology, Hanyang University ERICA Campus, Ansan 15588, Korea

***Correspondence to**

Daeho Park

School of Life Sciences

Gwangju Institute of Science and Technology

123 Cheomdangwagi-ro, Buk-gu

Gwangju 61005, Korea

Tel.: 82-62-715-2890

Fax: 82-62-715-2484

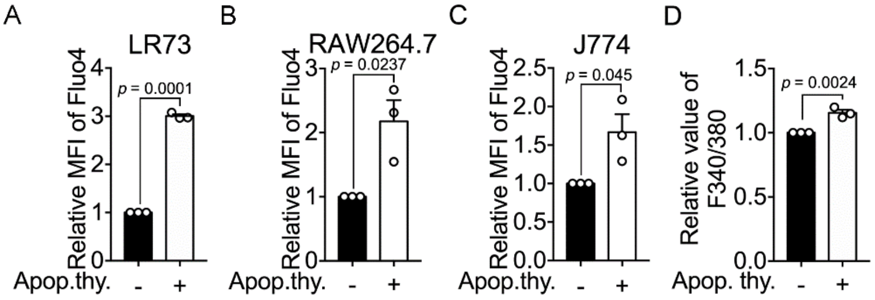

Figure S1: Elevation of the calcium level in phagocytes during efferocytosis

A, **B**, **C**. Fluo4-stained LR73 cells (**A**), Raw264.7 cells (**B**) or J774 cells (**C**) were incubated with or without apoptotic cells and analyzed by flow cytometry. The MFIs of Fluo4 in the cells were compared. n=3 experiments, mean ± SEM (two-tailed unpaired Student’s t test). **D**. Fura2-stained BMDMs were incubated with or without apoptotic cells and analyzed using a microplate reader. n=3 experiments, mean ± SEM (two-tailed unpaired Student’s t test).

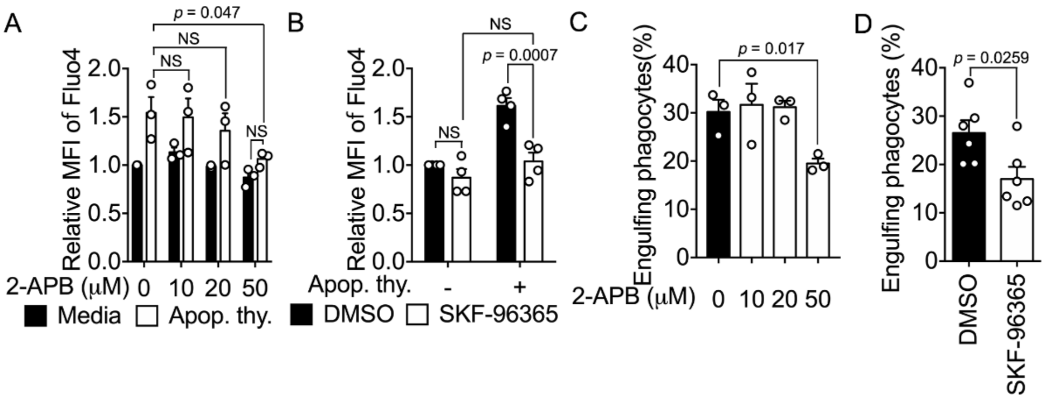

Figure S2: The effects of 2-APB and SKF-96365 on efferocytosis and the calcium elevation in phagocytes

A, **B**. Fluo4-stained BMDMs were incubated with apoptotic cells in the presence or absence of 2-APB (**A**, n=3 experiments) or SKF-96365 (**B**, n=4 experiments). The MFIs of Fluo4 in BMDMs were measured by flow cytometry. Mean ± SEM. NS, not significant (two-way ANOVA). **C**, **D**. BMDMs were incubated with TAMRA-stained apoptotic cells for 30 min in the presence or absence of 2-APB (**C**, n=3 experiments) or SKF-96365 (**D**, n=6 experiments) and analyzed by flow cytometry. mean ± SEM (two-tailed unpaired Student's t test).

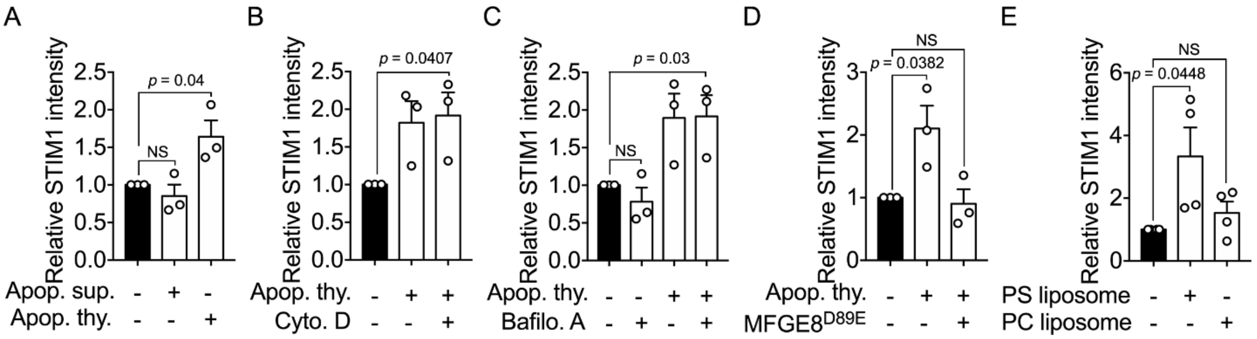

Figure S3: Quantification of immunoblots in Figure 4

A, **B**, **C**, **D**. LR73 cells transfected with STIM1 and Orai1-FLAG were incubated with apoptotic cells or apoptotic cell supernatants (**A**, n=3 experiments), with apoptotic cells in the presence or absence of cytochalasin D (**B**, n=3 experiments), bafilomycin A (**C**, n=3 experiments), or MFGE8^{D89E} (**D**, n=3 experiments), or with PC or PS liposomes (**E**, n=4 experiments) for 10 min. Orai1-FLAG in cell lysates was precipitated with anti-FLAG antibody-conjugated agarose beads. Bound proteins were detected by immunoblotting and co-immunoprecipitated STIM1 with Orai1 were quantified using ImageJ. Mean ±SEM (two-tailed unpaired Student's t test).

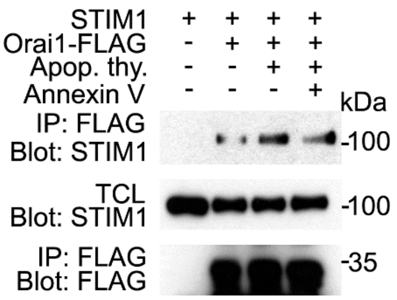

Figure S4: Blocking PS on apoptotic cells attenuates Orai1-STIM1 association

LR73 cells transfected with the indicated plasmids were stimulated with apoptotic cells for 10 min in the presence or absence of Annexin V. Orai1-FLAG in cell lysates was precipitated with anti-FLAG antibody-conjugated agarose beads. Bound proteins were detected by immunoblotting.
